# Molecular and biochemical characterization of the NS1 protein of non-cultured influenza B virus strains circulating in Singapore

**DOI:** 10.1099/mgen.0.000082

**Published:** 2016-08-25

**Authors:** Muhammad Raihan Jumat, Puisan Wong, Raphael Tze Chuen Lee, Sebastian Maurer-Stroh, Boon Huan Tan, Richard J. Sugrue

**Affiliations:** ^1^​ School of Biological Sciences, Nanyang Technological University, 60 Nanyang Drive, 637551, Singapore; ^2^​ Detection and Diagnostics Laboratory, DSO National Laboratories, 27 Medical Drive, 117510, Singapore; ^3^​ Bioinformatics Institute (BII) 30 Biopolis Street #07-01, Matrix Building, 138671, Singapore

**Keywords:** Influenza B, NS1, Cleavage, Proteolysis, B/Lee/40

## Abstract

In this study we compared the NS1 protein of Influenza B/Lee/40 and several non-cultured Influenza B virus clinical strains detected in Singapore. In B/Lee/40 virus-infected cells and in cells expressing the recombinant B/Lee/40 NS1 protein a full-length 35 kDa NS1 protein and a 23 kDa NS1 protein species (p23) were detected. Mutational analysis of the NS1 gene indicated that p23 was generated by a novel cleavage event within the linker domain between an aspartic acid and proline at amino acid residues at positions 92 and 93 respectively (DP_92–93_), and that p23 contained the first 92 amino acids of the NS1 protein. Sequence analysis of the Singapore strains indicated the presence of either DP_92–93_ or NP_92–93_ in the NS1 protein, but protein expression analysis showed that p23 was only detected in NS1 proteins with DP_92–93._. An additional adjacent proline residue at position 94 (P_94_) was present in some strains and correlated with increased p23 levels, suggesting that P_94 _has a synergistic effect on the cleavage of the NS1 protein. The first 145 amino acids of the NS1 protein are required for inhibition of ISG15-mediated ubiquitination, and our analysis showed that Influenza B viruses circulating in Singapore with DP_92–93 _expressed truncated NS1 proteins and may differ in their capacity to inhibit ISG15 activity. Thus, DP_92–93_ in the NS1 protein may confer a disadvantage to Influenza B viruses circulating in the human population and interestingly the low frequency of DP_92–93_detection in the NS1 protein since 2004 is consistent with this suggestion.

## Data Summary

1. Nucleotide sequences of 36 Influenza B NS1 from Singaporean clinical specimens have been deposited in GenBank; accession number: KC844161-KC844196 (url- http://www.ncbi.nlm.nih.gov/popset/?term=KC844161-KC844196).
2. Nucleotide sequences of the representative strains and their mutants have been deposited in GenBank; accession numbers: KC844195.1, KU500812, KC844165.1, KC844167.1, KC844163.1 (url- http://www.ncbi.nlm.nih.gov/nuccore/KC844195.1 http://www.ncbi.nlm.nih.gov/nuccore/KU500812 http://www.ncbi.nlm.nih.gov/nuccore/KC844165.1 http://www.ncbi.nlm.nih.gov/nuccore/KC844167.1 http://www.ncbi.nlm.nih.gov/nuccore/KC844163.1).

## Impact Statement

We report a novel cleavage event observed in a subset of Influenza B NS1 proteins. This cleavage is dependent on the presence of amino acids DP at positions 92–93. This event cleaves the NS1 protein in the linker domain, separating the RNA binding domain from the effector domain. The smaller protein containing the RNA binding domain has all the residues crucial for RNA binding and the nuclear localization signal, but this cleavage activity interrupts the amino acid sequences which are responsible for ISG15 inhibition. We believe that viruses with the cleavage motif would have altered viral fitness, evidenced by the temporal expression of such viruses over the last 30 years. We propose the possibility of the NS1 protein undergoing autoproteolytic cleavage as this cleavage was uninhibited by host cell proteases. The data presented in this study suggests a novel post-translational modification of the Influenza B NS1 protein, which may have significant effects on viral replication and the inhibition of host cell response.

## Introduction

Both Influenza A and B viruses contribute to the annual epidemic infecting a large percentage of children and the immunologically compromised, resulting in an annual death toll of up to 500,000 deaths (World Health Organization, 2014). While not studied as extensively as Influenza A, Influenza B virus infection can also lead to severe symptoms (Derlet, 2010; Kim *et al.*, 2009; Wu *et al.*, 2009; Michael *et al.*, 1980; Baine *et al.*, 1980). The Influenza B virus non-structural protein 1 (NS1) counters the host’s antiviral response and therefore represents a determinant of pathogenicity (Dauber *et al.*, 2004; Talon *et al.*, 2000; Sridharan *et al.*, 2010; Yuan & Krug, 2001). Influenza B virus induces interferon signalling following infection, leading to the induction of several interferon stimulatory gene proteins that induce an antiviral state in the infected cell (Randall & Goodbourn, 2008). Among these is the interferon-induced ubiquitin-like ISG15 protein, which covalently attaches via its C-terminal glycine to several target proteins, inducing the degradation of these cellular proteins (reviewed in Zhao *et al.*, 2013). The Influenza virus B virus NS1 protein inhibits the conjugation of the ubiquitin-like ISG15 protein to its target proteins (Yuan & Wang, 2001), suggesting that this is a factor in overcoming the host antiviral response. Interestingly, the interaction between NS1 and the ISG15 protein is species-specific, binding only human and non-human primate ISG15 (Sridharan *et al.*, 2010).

The NS1 protein is 281 amino acids (aa) long (Briedis & Lamb, 1982; S, 2013) and consists of two domains. The first 90 amino acid residues at the N-terminal end of this protein constitute the RNA binding domain (RBD) (Guan *et al.*, 2011). The RBD has been shown to bind to double-stranded RNA (Dauber *et al.*, 2006), contains the nuclear localization signal (NLS) (Schneider *et al.*, 2009), and is crucial for the dimerization of the NS1 protein (Wang & Krug, 1996). Although the full structure of Influenza B NS1 is yet to be elucidated, the structure of the first 120 amino acids has been determined (Li *et al.*, 2011). The effector domain (ED) of the NS1 protein is formed by amino acids 120–281, but no specific function has been assigned to this domain (Donelan *et al.*, 2004; Wang & Krug, 1996). These two domains are connected via a stretch of amino acids that constitutes the linker (L) region (Guan *et al.*, 2011).

The nature of this interaction between the NS1 and ISG15 proteins has been established biochemically. Although ISG15 binding activity in the NS1 protein resides in the RBD and part of the L region, inhibiting the conjugation activity of the ISG15 protein requires the first 145 amino acid residues (Yuan & Krug, 2001). This includes a large portion of the ED. Interestingly, Influenza virus B isolates in which the carboxyl terminal domain of the NS1 protein is deleted (e.g. Tobita *et al.*, 1990) are unable to inhibit the ubiquitination activity of the ISG15 protein in virus-infected cells and replicate less efficiently than viruses containing a full-length NS1 sequence (Yuan & Krug, 2001). Therefore, the presence the RBD, L and part of the ED is required for the ISG15 inhibitory activity of the NS1 protein.

In this study, we examined the expression of the NS1 protein in several non-tissue-culture-adapted Influenza B viruses that were detected during routine influenza surveillance in Singapore over the period 2004–2009 (Jumat *et al.*, 2014). Since these virus strains could not be cultured in tissue culture we have relied on recombinant expression of the cloned sequences. In this context, we have compared the expression profiles of the NS1 proteins derived from these clinical strains proteins with the recombinant NS1 protein of the established influenza virus B laboratory isolate B/Lee/40 and the NS1 protein expressed in Influenza B/Lee/40-infected cells. We provide the first report, to our knowledge, of the identification of a novel sequence motif (DP_92–93_) in the NS1 protein of the influenza virus B laboratory isolate B/Lee/40 that leads to a post-translational cleavage event. Furthermore, this modification is also observed in some Influenza B virus strains that are circulating in Singapore, suggesting clinical relevance.

## Methods

### Cloning of NS1 genes from throat swabs.

Throat swabs were collected from Singapore Armed Forces (SAF) servicemen displaying respiratory symptoms and were tested for Influenza B infection as described previously (Jumat *et al.*, 2014). Out of 88 throat swabs, 46 tested positive for Influenza B virus. To attain the sequence of the NS1 gene, gene segment 8 was amplified twice using four separate primers, producing overlapping reads, targeting the open reading frame of NS1 (Jumat *et al.*, 2014). Sequences were assembled using Seq-Man Program (DNASTAR). Out of these 46 strains, 36 NS1 complete gene sequences were obtained. These sequences were grouped according to sequence similarity (GenBank accession numbers KC844161–KC844196). Representative strains from each of these groups (DSO_090136_2004: KC844195.1, DSO_040117_2006: KU500812, DSO_010147_2007: KC844165.1, DSO_020132_2007: KC844167.1 and DSO_0070_2009: KC844163.1) were PCR-amplified using the primers NS1-F (Sac1) and NS1-R (XhoI) (Table S1, available in the online Supplementary Material) and ligated into the mammalian expression vector pCAGGS between the Sac1 and XhoI restriction sites (Patch *et al.*, 2007). Individual domains were PCR amplified similarly, by using the primers specific for each domain (Table S1). Two strains were chosen to represent specimens from 2007; DSO_020132_2007 and DSO_010147_2007, as DSO_020132_2007 represent the majority of the sequences isolated in 2007 and DSO_010147_2007 was unique to the other sequences isolated in 2007 (Jumat *et al.*, 2014).

### Site-directed mutagenesis.

DNA substitutions were introduced into the cloned gene segments by the use of the QuikChange Site-Directed Mutagenesis Kit (Stratagene) and in accordance to manufacturer’s instructions. Primers were designed with the substitutions incorporated through the use of the manufacturer’s online primer design tool (http://labtools.stratagene.com/QC). Table S2lists the primers used for mutagenesis experiments in this study. Generated mutants were sent to 1^st^ Base (Singapore) for DNA sequencing to confirm for the incorporation of the desired mutation.

### Antibodies.

The anti-FLAG and anti-Myc antibodies were purchased from Sigma-Aldrich and Cell Signaling respectively. The Influenza B NS1 antibody was a gift from Professor Thorsten Wolff from the Robert Koch Institut (Dauber *et al.*, 2004).

### Influenza B Infection.

Human embryonic kidney (HEK) 293T cells were infected with B/Lee/40 strain (ATCC VR101) at a multiplicity of infection (MOI) of 3, at 37 ˚C for 1 h. The inoculum was replaced with DMEM (GIBCO) + 2 % FBS (GIBCO) for 20 h before further analysis.

### Expression of NS1 constructs.

HEK 293T cells were transfected with the NS1-pCAGGS constructs using Lipofectamine 2000 reagent (Invitrogen) following the manufacturer’s instructions. The media was replaced at 4 h after transfection and continued to be incubated at 37 ˚C for 16 h before further analysis.

### Western blotting.

Cells expressing the protein of interest were scraped in 50 µl of 1× Laemmli Buffer and boiled at 95 ˚C for 10 min. Samples were then sonicated briefly and equal volumes were loaded onto SDS-PAGE gels. Protein bands were then transferred onto PVDF membranes (Immobilon-P, Millipore). Protein bands were then probed with anti-FLAG, cMyc and Influenza B NS1 proteins as appropriate. The proteins bands were visualised using ECL (GE Healthcare). Molecular size estimations were established by plotting the R_f_ of the All Blue (BioRad) molecular weight standards (distance migrated by specific band/ distance migrated by dye front) by the Log value of the molecular weight (Log MW). A best-fit line was obtained for each blot and the equation of the line was used to estimate the Log MW of the protein bands observed.

### Global frequencies of residues.

Protein sequences of human NS1 from influenza B viruses were downloaded from GISAID’s EpiFlu and NCBI’s Genbank databases. Sequences from both databases were merged and repeated strains were removed. MAFFT (http://www.ncbi.nlm.nih.gov/pubmed/15661851) was used for sequence alignment and global frequencies of residues were confined to strains from 1980 onwards. Geographical locations of strains were parsed from their strain names and the Google Static Maps API was used to generate the global geographical distribution of strains on a yearly basis.

## Results

### Detection of a novel NS1-related protein species in influenza B virus-infected cells

**Fig. 1.**
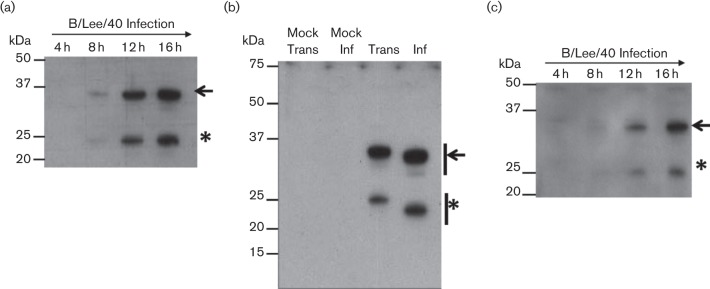
SDS–PAGE analysis of influenza B NS1 protein. (a). Time course infection of B/Lee/40 on HEK 293T cells. Cells were harvested at 4, 8, 12 and 16 h post infection and immunoblotted with αNS1. (b). HEK 293T cells were infected with B/Lee/40 virus (inf) or transfected with pCAGGS/NS1B-FLAG (Trans) and after 20 h the cell lysates were immunoblotted with αNS1. Mock Trans: Cells transfected with empty pCAGGS vector. Mock Inf: non-infected cells. (c). pCAGGS/NS1B-FLAG-transfected HEK 293T cell lysates were harvested at 4, 8, 12 and 16 h post transfection and immunoblotted using anti-FLAG. The full length (black arrow) and smaller (*) NS1 proteins are indicated.

We first examined the expression of the B/Lee/40 NS1 protein in virus-infected cells and in cells expressing the recombinant B/Lee/40 NS1 protein. MDCK cells were infected with B/Lee/40 and at 4, 8, 12 and 16 h post infection (h.p.i.) cell lysates were prepared in Laemmli buffer and the NS1 protein analysed by immunoblotting using Influenza B NS1 antibody (αNS1) (Fig. 1a). At 8 h.p.i. a 35 kDa protein species of the expected size for the NS1 was detected, together with a smaller 23 kDa protein species. The two protein species were detected concomitantly, and the levels of both proteins increased with increasing incubation time. This suggested the presence of a post-translational modification that led to the generation of a second NS1 protein species.

To determine if this was specific to virus-infected cells, we also examined the expression of the recombinant B/Lee/40 NS1 protein. The B/Lee/40 NS1 gene was cloned (with a FLAG-tag coding sequence introduced at the 3′ end of the NS1B gene) and inserted into the mammalian expression vector pCAGGS to generate pCAGGS/NS1B(LEE)-FLAG. HEK 293T cells were either infected with B/Lee/40 or transfected with pCAGGS/NS1B(LEE)-FLAG and at 16 h.p.i. or 16 h post transfection (h.p.t.) respectively cell lysates were prepared and examined by immunoblotting using αNS1 (Fig. 1b). In pCAGGS/NS1B(LEE)-FLAG transfected cells, two species of approximately 35 kDa and 24 kDa were observed, which migrated slightly more slowly than the corresponding protein species detected in the virus-infected cells. This difference in migration was due to the presence of the FLAG epitope tag added to the C-terminus on the recombinant expressed NS1 protein. No protein bands were observed in either the mock-infected or mock-transfected cell lysates, thus demonstrating the specificity of αNS1. Similarly, HEK 293T cells were transfected with pCAGGS/NS1B(LEE)-FLAG and at intervals between 4 and 16 h.p.t. cell lysates were prepared and immunoblotted using anti-FLAG (Fig. 1c). A concomitant increase in the appearance of both protein species was observed from 8 h.p.t., which is consistent with the kinetics of NS1 gene expression observed in virus-infected cells described above.

Although the second smaller NS1 protein species is observed during Influenza B virus infection, it is also seen during NS1 protein expression in the absence of virus infection. This suggests that this is an intrinsic property of the B/Lee/40 NS1 protein, and does not arise due to an antivirus response during influenza B virus infection. In this study, the full-length and smaller NS1 protein species will be referred to as the NS1 and p23 proteins respectively.

### p23 is composed of the effector domain and part of the linker region

**Fig. 2.**
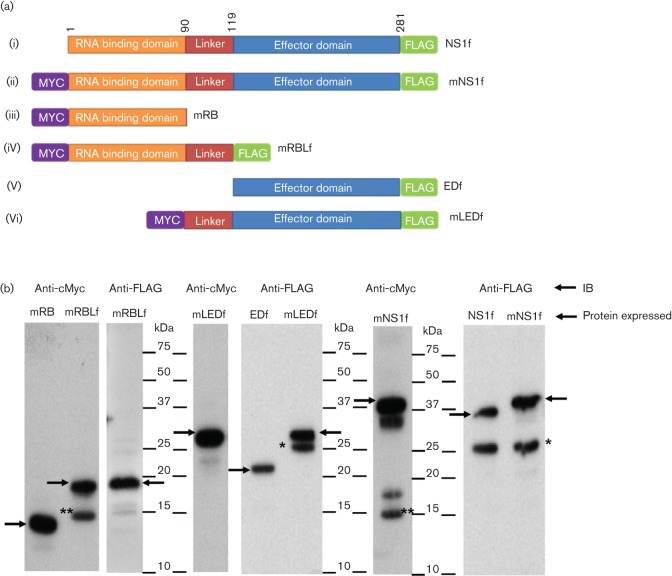
Expression analysis of individual NS1 domains. (a). Schematic representation of the full-length NS1 protein and separate domains of Influenza B/Lee/40 NS1 which were cloned into pCAGGS vector with N-terminal cMyc (m) and C-terminal FLAG(f) epitope tags. (i) NS1f, (ii) mNS1f; (iii) mRB (; (iv) mRBLf; (v) EDf and (vi) mLEDf. Numbers at the top of the diagram indicate amino acid sequence positions. (b). At 16 h post transfection HEK 293T cells expressing each of the recombinant NS1 proteins indicated were immunoblotted with anti-Myc and anti-FLAG antibodies as appropriate. Protein bands corresponding to the full-length expressed protein (black arrow), p15 (**) and p23 (*) are indicated. NS1, full-length protein; RB, RNA binding domain; RBL, RNA binding domain with linker; ED, effector domain; LED, linker with effector domain.

Mutational analysis of recombinant B/Lee/40 NS1 protein was performed to determine the identity of p23. A series of plasmid constructs were produced in which both full-length and individual domains of the B/Lee/40 NS1 protein were expressed with N-terminal cMyc- or C-terminal FLAG epitope tags as appropriate (Fig. 2a). These were full-length NS1B-FLAG (NS1f), full-length cMyc-NS1B-FLAG (mNS1f), cMyc-RBD (mRB), cMyc-RBD-L-FLAG (mRBLf), ED-FLAG (EDf), and cMyc-L-ED-FLAG (mLEDf) (Fig. 2a). Cells expressing each of these protein species were examined by immunoblotting with anti-cMyc or anti-FLAG as appropriate.

In cells expressing NS1f, immunoblotting with anti-FLAG showed protein species corresponding in size to NS1-FLAG and p23-FLAG (Fig. 2b). In cells expressing mNS1f and immunoblotted with anti-FLAG, only p23-FLAG and NS1-FLAG was detected, while immunoblotting with anti-cMyc revealed cMyc-NS1-FLAG and a protein species of approximately 15 kDa (p15). The p15 was also detected in cells expressing mRBLf and immunoblotted with anti-cMyc but was not detected when immunoblotted with anti-FLAG (Fig. 2b). The RBD, composed of 90 amino acids (Guan *et al.*, 2011), was estimated *in silico* to be 13 kDa (by BioEdit 7.2.5 software), therefore the immunoblotting analysis suggested that this domain is present within p15 (Fig. 2b). Immunoblotting using either anti-cMyc or anti-FLAG revealed a major protein species of 29 kDa. A smaller product was observed by immunoblotting with anti-FLAG that was not observed by immunoblotting with anti-cMyc, suggesting that this smaller product may have lost the cMyc tag. In comparison, the cells expressing EDf and immunoblotted with anti-FLAG revealed a major protein species of 22 kDa. A comparison of the sizes of these individual domains with the p23 observed in cells expressing NS1f and mNS1f suggested that p23-FLAG is composed of part of the linker region and the whole of the ED, while p15 is composed of the RBD and part of the linker region.

### Aspartic acid at position 92 in clinical strains correlates with the presence of p23

**Fig. 3.**
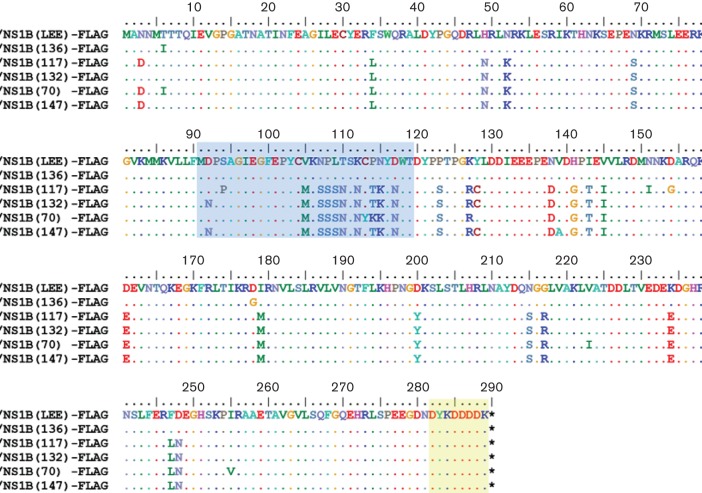
Amino acid sequence alignment of influenza B NS1 proteins used in this study. The coding sequences of the NS1 gene from B/Lee/40 (NS1B(LEE)-FLAG) and clinical specimens DSO_090136_2004 (NS1B(136)-FLAG); DSO_040117_2006 (NS1B(117)-FLAG); DSO_020132_2007 (NS1B(132)-FLAG DSO_010147_2007); DSO_0070_2009 (NS1B(70)-FLAG) and DSO_010147_2007 (NS1B(147)-FLAG). A FLAG epitope tag at the C-terminus (highlighted in yellow) and linker domain (amino acids 91–119) (highlighted in blue) are indicated. (.) Indicates identical sequence to NS1B(LEE)-FLAG. (*) correspond to the stop codon in the nucleotide sequence.

Previous examination of the predicted protein sequences of the NS1 proteins of Influenza B viruses detected in Singapore between 2004 and 2009 revealed that the specimens phylogenetically clustered according to their year of isolation (Jumat *et al.*, 2014). These clinical specimens were sequenced directly without prior culturing in either eggs or tissue culture and hence avoided mutations due to culture selection (Jumat *et al.*, 2014). We were therefore, interested to determine if sequence variation between clusters would result in differences in the corresponding biochemical properties in the NS1 protein. The expression of the NS1 gene of representative strains from each cluster was therefore examined, and five representative virus strains were selected for further analysis since these were predicted to exhibit sequence variation in the linker region of the corresponding NS1 protein. These were DSO_090136_2004 (136), DSO_040117_2006 (117), DSO_020132_2007 (132), DSO_0070_2009 (70) and DSO_010147_2007 (147). The NS1 genes from these strains were cloned into pCAGGS containing a FLAG tag at the C-terminus of the NS1 protein to aid protein detection (Fig. 3).

**Fig. 4.**
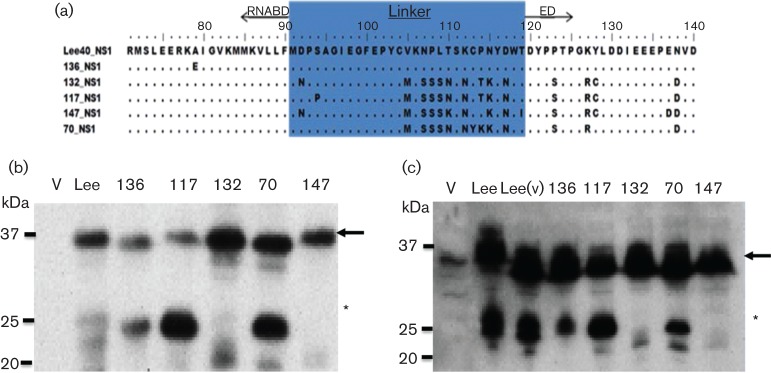
Expression of NS1 protein from five clinical specimens. (a). Amino acid alignment of NS1B(LEE)-FLAG (Lee-NS1), NS1B(136)-FLAG (136-NS1), NS1B(117)-FLAG (117-NS1), NS1B(132)-FLAG (132-NS1), NS1B(70)-FLAG (70-NS1) and NS1B(147)-FLAG (147-NS1) at amino acid positions 70–140. The linker domain (highlighted in blue) and the flanking RNA binding domain (RNABD) and effector domain (ED) are highlighted (b and c). Expression of the NS1 constructs in HEK 293T cells. At 16 h post transfection cells expressing NS1B(LEE)-FLAG, NS1B(136)-FLAG, NS1B(117)-FLAG, NS1B(132)-FLAG, NS1B(70)-FLAG and NS1B(147)-FLAG were immunoblotted with (b) anti-FLAG antibody or (c) αNS1 Cells transfected with empty pCAGGS Vector (V) or infected with Influenza B/Lee/40 (Lee(v)) are indicated. The NS1 protein (black arrows) and p23 (*) are highlighted.

This generated pCAGGS/NS1B(136)-FLAG, pCAGGS/NS1B(117)-FLAG, pCAGGS/NS1B(132)-FLAG, pCAGGS/NS1B(70)-FLAG and pCAGGS/NS1B(147)-FLAG (Fig. 4a). HEK 293T cells were transfected with each of these expression plasmids, together with pCAGGS/NS1B(LEE)-FLAG, and at 20 h.p.t. the cell lysates were examined by immunoblotting with anti-FLAG antibody. The p23-FLAG protein was detected in cells expressing NS1B(LEE)-FLAG, NS1B(136)-FLAG, NS1B(117)-FLAG and NS1B(70)-FLAG, while we failed to detect p23 in NS1B(132)-FLAG or NS1B(147)-FLAG-transfected cells (Fig. 4b). Similarly, when probed with αNS1 the p23-FLAG was not detected in NS1B(132)-FLAG or NS1B(147)-FLAG expressing cells (Fig. 4c).

**Fig. 5.**
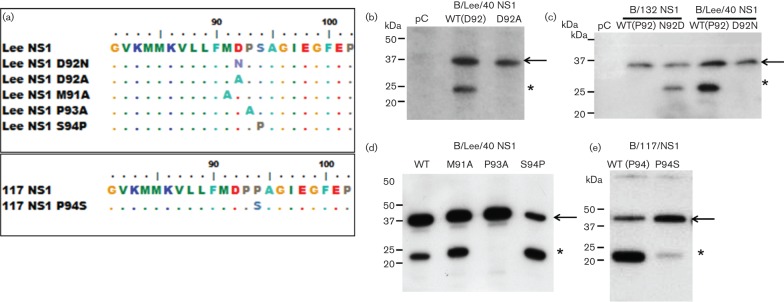
Mutational analysis of Influenza B NS1 protein. (a). Sequence alignment (between amino acids 81 and 102) of Lee-NS1 and the mutants Lee-NS1 D92N, Lee-NS1 D92A, Lee-NS1 M91A, Lee-NS1 P93A and Lee-NS1 S94P. Also shown are 117-NS1 and 117-NS1 P94S. (B–E). Protein expression profile of (b) Lee-NS1 wild-type (WT) and mutant Lee-NS1 D92A, (c). 132-NS1 wild-type (WT), 132-NS1 N92D, Lee-NS1 wild-type (WT) and Lee-NS1 D92N, (d). Lee-NS1 wild-type (WT), Lee-NS1 M91A, Lee-NS1 P93A, Lee-NS1 S94P and (e). 117-NS1 wild-type (WT) and 117-NS1 P94S. At 16 h post transfection, NS1 protein was detected by immunoblotting with anti-FLAG. pC: Cells transfected with empty pCAGGS vector. Protein bands corresponding to NS1-FLAG (black arrow) and p23-FLAG (*) are indicated.

Sequence analysis indicated that p23-FLAG was detected in clinical strains that had an aspartic acid at position 92 (D_92_), while p23-FLAG was not present in NS1B(132)-FLAG and NS1B(147) that contained an asparagine at this position (N_92_) (Fig. 4a). This suggested that the presence of D_92_ was a major determinant for the presence of p23. To confirm the role of D_92_ in generating p23, mutational analysis of the linker region in the B/Lee/40 NS1 protein sequence was performed. A series of mutants in the linker region in NS1 protein coding region within pCAGGS/NS1B(LEE)-FLAG, was constructed to generate D92A and D92N. In addition, the amino acid residues immediately adjacent to D_92_ were also mutated to M91A and P93A. These site-directed mutations gave rise to the mutated NS1(D92A), NS1(D92N), NS1(M91A) and NS1(P93A) proteins (Fig. 5a).

p23-FLAG could not be detected in cells expressing either NS1(D92A) or NS1B(D92N), confirming that D_92_ was required to generate p23 (Fig. 5b). Examination of the clinical strains NS1B(132)-FLAG and NS1B(147)-FLAG showed that the presence of N_92_ in the 132-NS1 sequence correlated with the absence of p23-FLAG. Therefore, a reverse mutation (N92D) was introduced in 132-NS1 and the protein examined by immunoblotting within anti-FLAG. This amino acid substitution correlated with the presence of p23-FLAG in this mutant (Fig. 5c). In cells expressing Lee-NS1(D92N), p23-FLAG was not detected even after longer expression times, suggesting that the D92N mutation prevented the appearance of p23-FLAG rather than delaying its appearance (results not shown).

Leaky scanning of the ATG leading to protein variation has been described for several Influenza virus proteins (Wise *et al.*, 2009; Tauber *et al.*, 2012; Muramoto *et al.*, 2013). To eliminate the possibility that p23 could arise due to leaky scanning of the ATG at M_91_, we substituted the methionine with an alanine, Lee-NS1 (M91A). p23 was still detected in cells transfected with Lee-NS1 (M91A) (Fig. 5d). This suggested that p23-FLAG did not arise to leaky scanning since there are no other methionine residues present in the linker domain of Lee-NS1 (Fig. 3).

### The adjacent proline residue at position 93 is required for p23 generation

Interestingly, the P93A substitution in Lee-NS1 sequence correlated with the absence of p23-FLAG detection, in a similar manner to cells expressing Lee-NS1 (D92N and D92A) (Figs. 5b & d), suggesting that in addition to D_92_, the adjacent P_93_ also is important for generating p23. In contrast to Lee-NS1 and the other clinical specimens analysed, the construct 117-NS1 showed a more intense p23-FLAG band compared with the full-length protein. Sequence comparison showed that in addition to N_92_ and P_93_, there was an additional proline at aa position 94 (P_94_), while in the other NS1 sequences a serine was present at this position. This suggested that the additional proline may facilitate for the presence of p23-FLAG. Therefore, the 117-NS1 was mutated to 117-NS1 (P94S) and its expression examined by western blotting. This substitution resulted in a reduced appearance of p23-FLAG (Fig. 5e). Similarly, mutating the Lee-NS1 sequence to introduce a proline at position 94 [Lee-NS1 (S94P)] resulted in the increased appearance of p23-FLAG (Fig. 5d). Collectively this analysis defined the amino acid sequence within the NS1 protein linker domain that gave rise to p23. The presence DP_92–93 _is required for NS1 protein cleavage, while a proline at position 94 facilitates the formation of p23.

### The NS1 p23 protein is only found in the cytoplasm

**Fig. 6.**
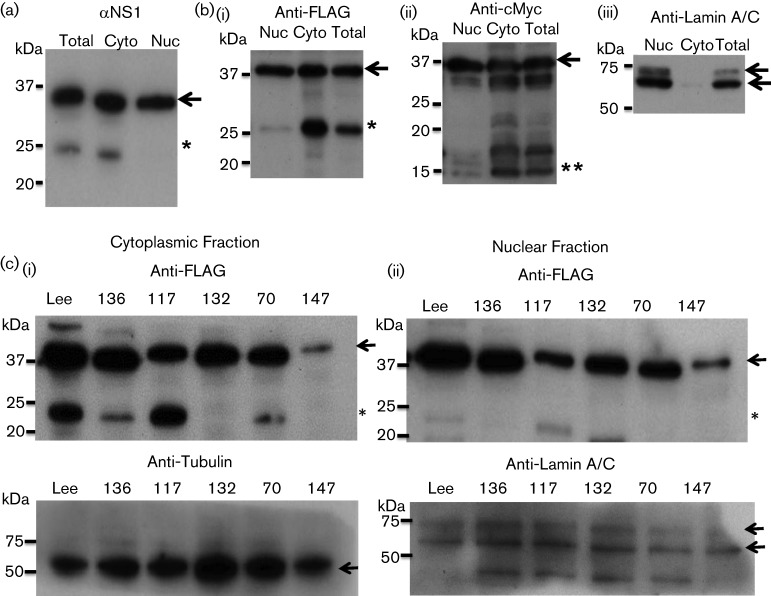
p23 is located in the cytoplasm of NS1-expressing cells. (a). HEK 293T cells were infected with influenza B/Lee/40 virus and at 20 h post infection the cells were fractionated into cytoplasmic (Cyto) and nuclear (Nuc) fractions. Total homogenate (Total) and the Cyto and Nuc fractions were then immunoblotted with αNS1. The full-length NS1 protein (black arrow) and p23 (*) are highlighted. (b) HEK 293T cells expressing c-yc-NS1(LEE)-FLAG were fractionated into cytoplasmic (Cyto) and nuclear (Nuc) fractions, and the total homogenate (Total), Cyto and Nuc fractions were immunoblotted using (i) anti-FLAG and (ii) anti-cMyc. The full-length NS1 protein (black arrows), p23 (*) and p15(**) are highlighted. (iii) Immunoblotting with anti-lamin A/C (nuclear marker). (c). Fractionation analysis of HEK 293T cells expressing Lee-NS1, 136-NS1, 117-NS1, 132-NS1, 70-NS1 and 147-NS1. Cytoplasmic and nuclear fractions are indicated. Immunoblotting was performed with anti-FLAG to detect the NS1 protein. Anti-tubulin (cytoplasmic marker) and anti-lamin A/C (nuclear marker) are loading controls for each fraction. The full lenght NS1 protein (black arrows) and p23 (*) are highlighted in immunoblotting using anti-FLAG. The location of Tubulin and Lamin A/C is also highlighted (black arrows) in immunoblotting using anti-Tubulin and anti-Lamin A/C respectively.

We fractionated B/Lee/40 NS1-infected cells and pCAGGS/cMYC-NS1B(LEE)-FLAG-transfected cells (Fig. 6) to determine where in the cell the p23 was generated. This would allow us to identify where in the cell the biological activity that gave rise to p23 was located. Examination of cell fractions prepared from virus-infected cells by immunoblotting using αNS1 revealed that p23 was only present in the cytoplasmic fraction (Fig. 6a). Examination of pCAGGS/cMYC-NS1B(LEE)-FLAG-transfected cells by immunoblotting using anti-FLAG and anti-cMyc revealed that p23 and p15 were only detected in the cytoplasmic fraction (Fig. 6b). A similar analysis of cells expressing the NS1 clinical strain also showed that p23 was detected in the cytoplasmic fraction (Fig. 6c). These observations suggest that the event leading to the transformation of the NS1 protein into p23 occurs in the cytoplasm.

### Evidence for negative selection of the N92D change in the NS1 protein of circulating Influenza B viruses

**Fig. 7.**
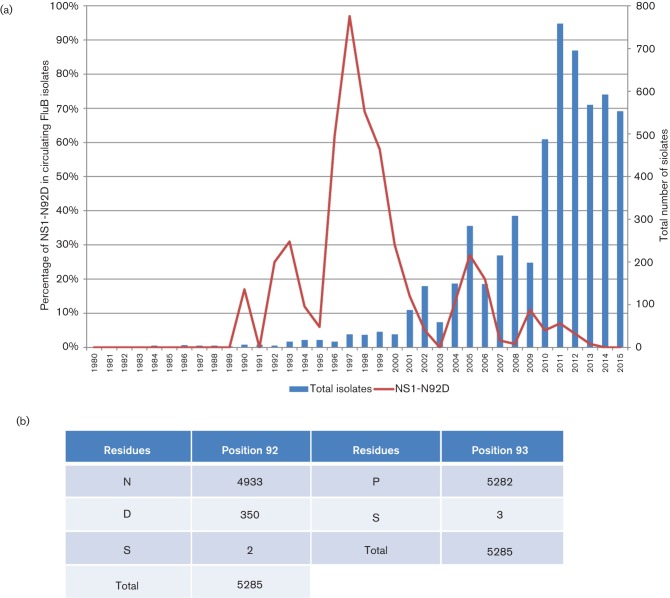
The frequency of the NS1 N92D substitution from 1980 to 2015 in influenza B viruses. (a) Frequency changes of the NS1 N92D substitution and the total count of isolates in each year from 1980 to 2015. (b) Global count of residues at positions 92 and 93 of NS1 from Influenza B viruses. Count of residues from all 5285 Influenza B sequences from 1940 to 2015. The N92D substitution represents a frequency of 6.6 % in all Influenza B NS1 sequences.

Examination of the data obtained in our previous analysis showed that all strains sequenced had either an N or D at position 92, of which N_92_ and D_92_ accounted for 14 % and 86 % of the strains respectively (Jumat *et al.*, 2014). However, our analysis was confined to Influenza B virus strains circulating in Singapore, and we were interested to determine the presene of this sequence motif in viruses circulating in other regions. We investigated the frequency of the Influenza B virus NS1 N92D and P93S sequence mutations in the global surveillance of Influenza B viruses to determine the incidence of this modification in circulating viruses. There was limited availability of influenza B virus NS1 sequences prior to the 1980s, and our analysis was therefore confined to sequences that were deposited since 1980 (Fig. 7a). The P93S residues is present only in three strains (B/South Auckland/2/2011, B/Perth/505/2013, B/Philippines/4/2014), which also contain N_92_. Interestingly, very early reference strains such as B/Lee/40 and B/GL/54 have D_92_. Overall, N92D occurred at least 350 times which represents a frequency of 6.6 % in all Influenza virus B NS1 sequences (Fig. 7b). The temporal occurrence of N92D was not random but was characterised by different peaks of occurrence with the most prominent being in 1997 where 97 % of strains carried the N92D mutation. Analysis of the geographical distribution of strains with this mutation in 1997 suggested frequent occurrence in the Americas and Asia, but interestingly less so in Europe. There were also minor occurrences in 1990, 1992, 1993 with a bias towards Europe and North America, although the numbers of total sequences for these time periods are rather low. Another larger peak of occurrence for N92D was noted in 2005 where it accounted for 27 % of all deposited sequences, with a strong geographical bias towards South East Asia and Oceania (mainly Australia and New Zealand). Despite the larger number of total sequences being sampled, there has been a gradual decline of N92D up to 2015. In summary, the temporally and geographically biased appearance patterns and the clear decline in frequency despite increased surveillance sampling suggest that strains with the NS1 N92D sequence motif are not particularly successful in the environment when compared with NS1 N_92_-containing variants.

## Discussion

In this study, we report a novel post-translational modification of the Influenza B virus NS1 protein that leads to the formation of a truncated NS1 protein in the cytoplasm of infected cells. Although the biological mechanism that leads to the generation of p23 is still uncertain, our analysis suggested that this event occurs within the linker region of the NS1 protein. An RNA splicing event within the viral mRNA that encodes the NS1 protein is unlikely since this would lead to the removal of the FLAG-tag recognition motif. Similarly, the involvement of an alternative reading frame within the linker region is unlikely, since removal of the single methionine residue within the linker region failed to prevent the generation of p23. However, we currently cannot rule out the possibility that p23 is generated by a mechanism that involves an alternative reading frame elsewhere within the NS1 mRNA. The mutational analysis provided evidence that p23 may be generated by proteolysis, and we had suggested that this may involve a protease activity in the cytoplasm. In this context we tested a range of different protease inhibitors (e.g. serine protease, aspartic protease and metalloproteinase); however, none of these were able to inhibit p23 formation (Jumat and Sugrue, unpublished observations). Interestingly, protein sequences that contain a DP amino sequence have been reported to be cleaved by caspase enzymes (Alnemri *et al.*, 1996; Luthi & Martin, 2007), and caspases cleave a range of Influenza virus proteins (e.g. NP and M2; (Richard & Tulasne, 2012; Zhirnov *et al.*, 1999, 2002). This hypothesis was tested using the pan-caspase inhibitor (Z-VAD-FMK), however, at all concentrations used the drug treatment failed to block p23 formation (Fig. S1). This suggests that that cleavage of the NS1 protein may involve either a novel unidentified cellular protease or that the NS1 protein may undergo autoproteolysis (i.e. it is independent of any host cell proteases). The capsid protein of Flock House Viruses (FHV) and Black Beetle Viruses (BBV) undergoes autoproteolytic cleavage mediated by an aspartic acid initiating a nucleophilic attack on an asparagine residue; resulting in hydrolysis of the peptide bond (Wery *et al.*, 1994; Hosur *et al.*, 1987). Understanding this process has been facilitated by the availability of the entire structure of the capsid proteins (Speir *et al.*, 2010). Although structural information is only available for the first 103 amino acids of the Influenza B NS1 protein (Guan *et al.*, 2011), this does however suggest that the linker region may be exposed as a surface loop structure and could be prone to proteolysis.

It has been established that the NS1 from Influenza B virus is a major factor in overcoming the host antiviral response by inhibiting the conjugation of the ubiquitin-like ISG15 protein to its target proteins (Yuan & Wang, 2001). Although ISG15-binding activity in the NS1 protein resides in the RBD and part of the linker region, a large portion of the ED is also required to facilitate this biological activity (Yuan & Krug, 2001). Influenza virus B isolates in which the carboxyl terminal domain of the NS1 protein was deleted were unable to inhibit the ubiquitination activity of the ISG15 protein in virus-infected cells (Yuan & Krug, 2001). In addition, viruses in which the ED is removed from the NS1 protein also replicate less efficiently than virus containing a full-length NS1 protein (Yuan & Krug, 2001). The clinical strains that we examined were not passaged through tissue culture or embryonated eggs, ensuring that the sequence motif leading to the formation of p23 did not arise due to either egg- or tissue-culture-adaptation. It is predicted that removal of the ED may have a significant effect on the biological activity of the NS1 proteins in clinical strains exhibiting NS1 cleavage. We would, therefore predict that several of the clinical strains detected in our study would exhibit impaired biological activity in the NS1 protein and may differ in their replication capabilities in the host. However, in these previous studies the ED was not expressed, while in the strains we examined the NS1 protein is cleaved and both the RBD and ED are present. It is, therefore unclear if the cleaved ED can act interact with the RDB to establish the effect on ISG15. Unfortunately, we were unable to recover the specific Influenza B viruses detected in our study, and so have not been able to define the biological activity of these NS1 variants in the context of an infectious virus. Although our analysis suggested a higher frequency of the D92 in the strains that we examined, the analysis of the Influenza B virus NS1 sequences that have been deposited in the NCBI database indicate that the incidence of D_92_ has declined.

Although there are several factors that determine the fitness of virus to be maintained in the environment, our sequence analysis suggests that cleavage of the NS1 protein does not provide a competitive advantage to Influenza B viruses in the natural environment. Interestingly, we have thus far failed to detect a similar excision of the NS1 protein of Influenza A laboratory isolates, suggesting that this phenomenon may be specific to influenza B viruses. Analysis of the NS1 protein sequences from our sequence collection would not have provided any obvious prediction that the NS1 protein would be cleaved into two protein species. Therefore, although sequence analysis can provide useful information about virus evolution, our analysis highlights the utility of examining the biological properties of individually expressed proteins from non-cultured clinical strains to provide a fuller understanding of virus evolution in the natural environment. The biological significance of the NP_92–93 _in the NS1 protein will require further examination using recovered clinical strains.

## Supplementary Data

233 Supplementary File 1
