# Supplementary material for: Molecular and biochemical characterization of the NS1 protein of non-cultured influenza B virus strains circulating in Singapore
Source: Microb Genom. 2016 Aug 25;2(8):e000082. doi: 10.1099/mgen.0.000082 (PMC5320597; doi:10.1099/mgen.0.000082)
Supplement: Supplementary file 1 [file mgen-02-82-s001.pdf]

Supplementary Figure 1

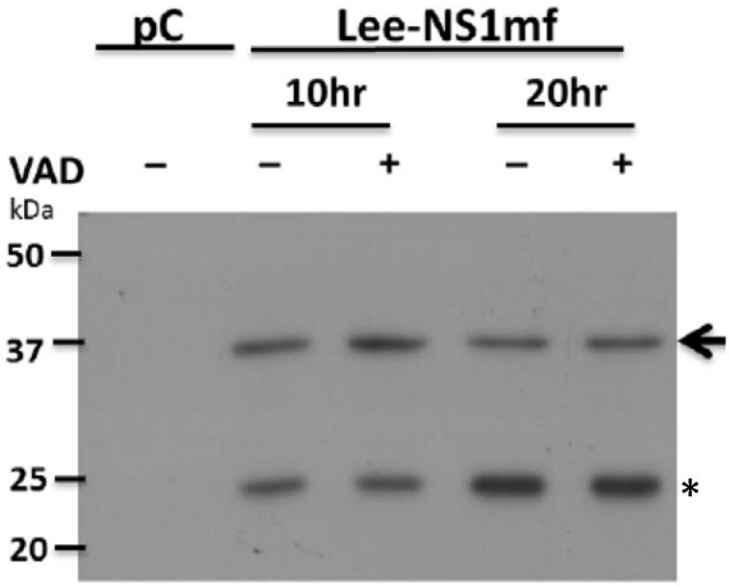

Supplementary Table 1

Primer Name	5' Sequence 3'
**Full Length**	
**NS1-For (Sac1):**	CGAGCTCGAAATG GCG RACAACATGAYC
**NS1-For Myc:**	CGAGCTCGAA ATGGAA CAA AAACTCATCTCAGAAGAGGAT CTG GCGRACAACATGAYC
**NS1-Rev FLAG (Xho1):**	CCGCTCGAGCGG CTATT TATC GTC ATC GTC TTT GTA ATCATTGTCTCC CTCTTCTGGTGA
**Individual Domains**	
**RB-Rev (Xho1):**	CCGCTCGAGCGG CTAAAAYAGRAGYACTTTCATCATT
**RBL-Rev (Xho1):**	CCGCTCGAGCGGCTATTTATCGTCATCGTCTTTGTAATCATCGGTCCAATCGTAATTTGGACATTT
**ED-For (Sac1):**	CGAGCTCGAAATGGATTAYCCTYCAACMCCAGGRARGTACC
**LED-For Myc (Sac1):**	CGAGCTCGAAATG GAACAAAAACTCATCTCAGAAGAGGATCTG
**LED-For (Sac1):**	CGAGCTCGAAATGGATCCCTCTGCTGGA

Supplementary Table 2

Primer Name<br>5' Sequence 3'	Specimen	Substitution
**Lee-NS1 M91A For:**<br>GGGTAAAAATGATGAAAGTGCTTCTGT TTGCGG ATCCCTCTGCTGG<br>**Lee-NS1 M91A Rev:**<br>CCAGCAGAGGGATCCGCAAACAGAAGCACT TTCATCATTTTTACCC	Lee-NS1	M91A
**Lee-NS1 D92N For:**<br>GAAAGTGCTTCTGTTTATGAATCCCTCTGCTGGAATTGAAGG<br>**Lee-NS1 D92N Rev:**<br>CCTTCAATTCCAGCAGAGGGATTCATAAACAGAAGCACTTTC	Lee-NS1	D92N
**Lee-NS1 D92A For:**<br>GAAAGTGCTTCTGTTTATG**GCT**CCCTCTGCTGGAATTGAAGG<br>**Lee-NS1 D92A Rev:**<br>CCTTCAATTCCAGCAGAGGGAGCCATAAACAGAAGCACTTTC	Lee-NS1	D92A
**Lee-NS1 P93A For:**<br>GAAAGTGCTTCTGTTTATGGATGCCTCTGCTGGAATT<br>**Lee-NS1 P93A For:**<br>AATTCCAGCAGAGGCATCCATAAACAGAAGCACTTTC	Lee-NS1	P93A
**Lee-NS1S94P For:**<br>CTTCTGTTTATGGATCCCCCTGCTGGAATTGAAGGAT<br>**Lee-NS1S94P Rev:**<br>ATCCTTCAATTCCAGCAGGGGGATCCATAAACAGAAG	Lee-NS1	S94P
**132-NS1 N92D For:**<br>GAAAGTACTCCTATTTATGGATCCGTCTGCTGGAATTGAAGGG<br>**132-NS1 N92D Rev:**<br>CCCTTCAATTCCAGCAGACGGATCCATAAATAGGAGTACTTTC	132-NS1	N92D
**117-NS1 P94S For:**<br>TCCTATTTATGGATCCGTCTGCTGGAATTGAAGGG<br>**117-NS1 P94S Rev:**<br>CCCTTCAATTCCAGCAGACGGATCCATAAATAGGA	117-NS1	P94S
